# Advances Toward a Norovirus Antiviral: From Classical Inhibitors to Lethal Mutagenesis

**DOI:** 10.1093/infdis/jiv280

**Published:** 2015-12-19

**Authors:** Lucy Thorne, Armando Arias, Ian Goodfellow

**Affiliations:** Division of Virology, Department of Pathology, University of Cambridge, Addenbrookes Hospital, United Kingdom

**Keywords:** human norovirus, antivirals, protease, polymerase, interferon λ, favipiravir, lethal mutagenesis

## Abstract

Human noroviruses are a leading cause of gastroenteritis worldwide, yet there are no licensed antivirals. There is an urgent need for norovirus therapeutics, particularly for chronic infections in immunocompromised individuals, but also a potential need for prophylactic use in epidemics. Continued research has led to the identification of compounds that inhibit norovirus replication in vitro and, at least in some cases, are also effective in vivo against murine norovirus. Progress has included classical approaches targeting viral proteins and harnessing the antiviral action of interferon, strategies targeting essential host cell factors, and novel strategies exploiting the high mutation rate of noroviruses.

Human noroviruses (HuNoVs) are a major cause of viral gastroenteritis worldwide, and since introduction of the rotavirus vaccine they have become the leading cause of severe pediatric gastroenteritis [1]. HuNoVs present a particular problem in healthcare settings, with close to 4000 hospital outbreaks reported in a 2-year period in the United Kingdom, causing approximately 9000 days of ward closures, disrupted services, and a significant economic cost for the UK National Health Service [2]. Most infections are acute with a low risk of mortality, but HuNoV is recognized as a significant risk factor for complications and increased mortality in immunocompromised individuals, including transplant recipients and those receiving immunosuppressive therapies [3]. In many cases, HuNoV infections in these cohorts can persist for years [4]. Conservative estimates of mortality rates in developing countries are much higher; at least 200 000 deaths per year in children aged <5 years are attributed to HuNoVs [5].

Despite an urgent need, there are currently no licensed antivirals or vaccines for HuNoVs. Groups that would most benefit include high-risk populations, such as young and elderly individuals, chronically infected patients, and medical staff. Healthcare and military settings could also benefit from prophylactic use of antivirals to potentially contain epidemics. However, the development of therapeutics has been hindered by the lack of an efficient cell culture system for HuNoV, which has also slowed elucidation of the molecular details of norovirus replication and viral protein functions, key knowledge for the rational development of specific antivirals. A replicon system, in which cells harbor self-replicating RNA of the prototype HuNoV, namely Norwalk virus, has provided an invaluable tool to evaluate antivirals in vitro [6]. In the past few years, however, there has been significant progress in the field, with the establishment of a plasmid-based reverse genetics system, to allow production of genetically defined HuNoVs, and the first demonstration of HuNoV replication in vitro in a cultured B-cell line and in vivo in immunodeficient mice, albeit with limited replication in both [7–9]. These developments may now provide systems to evaluate potential antivirals, but their usefulness will critically depend on being robust and reproducible in other laboratories.

Murine norovirus (MNV), which replicates in cultured macrophage and dendritic cells, has been used as a surrogate model for HuNoV since its discovery in 2003, owing to the availability of reverse genetics systems and small-animal models. Both HuNoV and MNV belong to the *Caliciviridae* family of small, positive-sense, single-stranded RNA viruses and have similar genome organization, protein functions, and conserved molecular mechanisms of genome replication and translation. By use of a combination of MNV and the HuNoV replicon, research efforts into antivirals have intensified in the last 10 years [6, 10]. This review will focus on the latest developments, which can be divided into categories of classical antiviral approaches that target viral proteins or harness the antiviral effects of interferon (IFN), alternative strategies targeting host cell processes, and strategies that exploit the high mutation rate of noroviruses by lethal mutagenesis.

## DEVELOPMENTS IN CLASSICAL ANTIVIRAL APPROACHES: RENEWED INTEREST IN IFN AND THE TARGETING OF VIRAL PROTEINS

Type I and type II IFNs elicit effective antiviral responses against HuNoV and MNV, emphasizing the critical role of innate immunity in controlling norovirus infections [6, 11]. Despite this, clinical use of IFN against HuNoVs has not been described. Interest in IFN as a HuNoV therapeutic has recently been renewed, owing to a study that found that type III IFN, IFN-λ, is required to control persistent MNV infections [11]. Treatment with IFN-λ cleared persistent infections in mice without requiring an adaptive immune response, revealing the potential of IFN-λ as a treatment for chronic infections in the immunocompromised.

**Figure 1.**
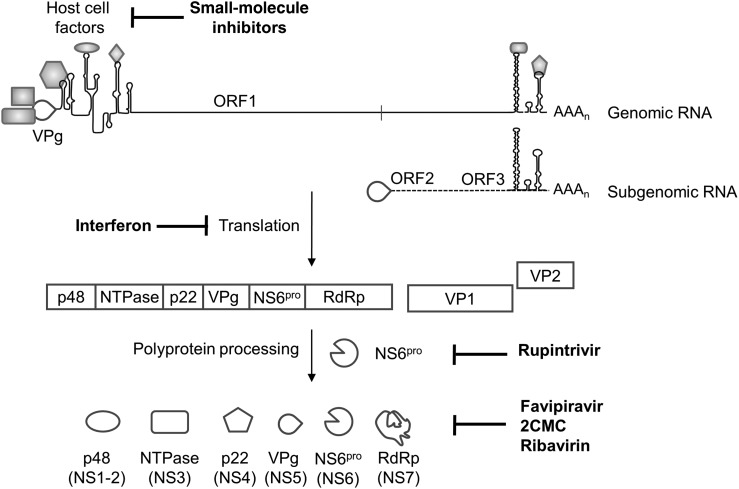
Organization of the human norovirus genome and the main antiviral targets. The genome is covalently attached at the 5′ end to VPg and is polyadenylated at the 3′ end. RNA structures are present at either end of the genome, which interact with host cell factors (shaded shapes) to achieve replication and translation. Essential host cell factors represent potential antiviral targets for small-molecule inhibitors. The viral genome is divided into 3 open reading frames (ORFs). ORF1 encodes the viral polyprotein, which is cotranslationally and posttranslationally cleaved by the viral protease, NS6^pro^, to release mature nonstructural proteins, including the viral RNA–dependent RNA polymerase (RdRp). Names given in brackets for the nonstructural proteins represent the alternative nomenclature used for murine norovirus. Both NS6^pro^ and the RdRp are key viral protein targets for a number of inhibitors as shown. ORF2 and ORF3 are translated from the subgenomic RNA and encode the major and minor capsid proteins respectively. The antiviral effects of type I and II interferons (IFNs) are thought in part to be mediated at the level of translation, although the mechanism of action of IFN-λ has not yet been determined. Abbreviation: 2CMC, 2′-C-methylcytidine.

A classical antiviral strategy is to target essential viral proteins, and for HuNoV the viral protease (NS6^pro^) has become the most widely studied antiviral target [10]. NS6^pro^ is a chymotrypsin-like cysteine protease responsible for cleavage of the viral polyprotein to release mature forms of the essential nonstructural replicase proteins, including itself (Figure 1). Resolution of the NS6^pro^ crystal structure in 2006 has since facilitated structure-guided design of a variety of inhibitors aimed to mimic natural substrate recognition and react irreversibly with active site residues. In the past 2 years, there has been a significant increase in the number of norovirus protease inhibitors that exhibit a range of potencies in in vitro enzymatic assays and cell-based assays [10]. NS6^pro^ shares similarities with the picornavirus protease (3C^pro^), and some compounds effective against 3C^pro^ exert broad reactivity against NS6^pro^. Most recently, rupintrivir, originally developed against the rhinovirus 3C^pro^, was found to clear cells of HuNoV replicon RNA and inhibit MNV replication in vitro. In enzymatic assays, rupintrivir inhibited the NS6^pro^ of the predominant circulating HuNoV genotype GII.4, suggesting that it may target clinically relevant strains [12].

Given their essential role in replicating the viral genome, viral RNA–dependent RNA polymerases (RdRps) also present attractive antiviral targets. Polymerase inhibitors have been clinically approved for many RNA viruses, whose RdRps share conserved structural and functional properties with the norovirus RdRp [13]. RdRp inhibitors can be divided into nucleoside analogues, which target the active site, and non-nucleoside analogues. The nucleoside analogue 2′-C-methylcytidine (2CMC), originally developed for use against hepatitis C virus (HCV), inhibits HuNoV replication in vitro, and treatment with 2CMC cleared cells of replicon RNA. 2CMC was also effective against MNV in vitro and in vivo*,* in which treatment prevented diarrhea and mortality in a lethal mouse model of infection [6, 10]. The same authors recently demonstrated that treatment with 2CMC reduced shedding and transmission of MNV and that prophylactic treatment completely protected against MNV infection [14]. While these results are highly promising, 2CMC has not yet been approved for treatment of HCV infection because of concerns regarding its toxicity profile in patients, although derivatives of 2CMC are currently under development and could prove of potential therapeutic use.

Several non-nucleoside inhibitors have been identified with activity against the HuNoV RdRp in enzymatic assays. These include suramin, NF203, and PPNDS [10]. Crystallographic studies have revealed the binding site of each compound on the RdRp, but they have yet to be tested in the cell culture or animal models of norovirus infections. Recently, a high-throughput screen against HuNoV GII.4 RdRp activity identified several compounds that also inhibited the Norwalk virus replicon and MNV replication in vitro, although at much higher concentrations. These compounds now provide a scaffold for rational design and optimization of specific HuNoV RdRp inhibitors [15].

## TARGETING THE HOST CELL AS AN ANTIVIRAL APPROACH

Owing to the high error rates of RNA virus replication, norovirus included, the efficacy of antivirals targeting viral proteins is often limited by the emergence of drug-resistance mutations. An alternative strategy that circumvents this problem is to target host cell proteins that are essential for viral replication, providing a much higher barrier to the generation of resistant mutants. A number of host cell factors have been identified that interact with the viral genome and are required for either its replication or translation; these include La, PTB, DDX3, PCPB2, and hnRNPs [6]. RNAi-mediated knockdown of these factors reduced MNV viral yields in vitro, demonstrating the potential of targeting host cell proteins to restrict replication. Modulation of entire cellular processes has also been shown as an antiviral approach [16]. WP1130, a small-molecule inhibitor of cellular deubiquitinases, inhibits replication of MNV and several other RNA viruses, with this activity indirectly mediated by activation of the unfolded protein response (UPR). Inhibition of a specific cellular deubiquitinase (UPS14) resulted in the activation of inositol-requiring enzyme, a key mediator of the UPR, in which endoplasmic reticulum–associated protein degradation is increased and cellular translation decreased. Small-molecule activators of the UPR also reduced MNV replication. Derivatives of WP1130 have recently been identified that have enhanced broad antiviral activity, without cellular toxicity, although they have yet to be evaluated in vivo [16].

## LETHAL MUTAGENESIS OF NOROVIRUSES

**Figure 2.**
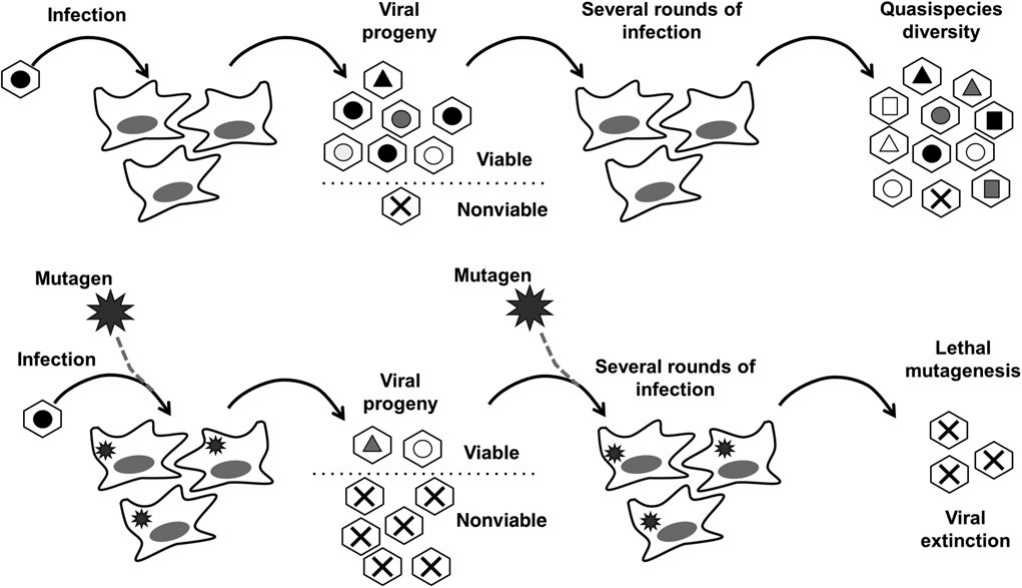
Lethal mutagenesis as an antiviral strategy to control norovirus. *Top*, During multiple rounds of virus infection in host cells, diverse virus populations (known as quasispecies) are formed as a result of low viral RNA–dependent RNA polymerase replication fidelity. Genetic diversity is represented as different virus particles containing different shape and color symbols. Some viruses will contain lethal mutations and are naturally non-viable, represented with a cross. Genetic diversity enables RNA viruses to have rapid adaptability to the environment and the flexibility to escape from immune responses and antiviral compounds. *Bottom*, Lethal mutagenesis exploits low replication fidelity to drive RNA viruses to extinction through an excessive accumulation of mutations. A mutagenic compound (black star) interferes with virus replication, leading to larger mutation frequencies. As a result of increased error rates during lethal mutagenesis, a larger proportion of viruses will contain lethal mutations. Continuous replication in the presence of an efficient mutagen can result in complete loss of viral infectivity (virus extinction).

Lethal mutagenesis has recently emerged as a novel alternative strategy to classical antiviral approaches. Several nucleoside analogues, such as ribavirin and favipiravir, display antiviral activities associated with induced mutagenesis in vitro [17, 18]. Excessive accumulation of mutations during replication, beyond a tolerated value known as the error threshold, can lead to virus extinction, a process known as lethal mutagenesis (Figure 2). The in vitro evidence has led to several studies that assessed the clinical use of lethal mutagenesis. Recent reports on HCV have suggested that the antiviral activity of ribavirin in vivo may be associated with increased mutagenesis, although this is still a controversial topic [18].

With the aim of assessing antiviral therapies based on lethal mutagenesis, we have recently investigated whether ribavirin and favipiravir are also mutagenic for noroviruses [17]. We found that treatment of MNV-infected mice with favipiravir resulted in more-rapid clearance rates of infectious virus. Viral populations from treated mice showed higher mutation frequencies and lower replication fitness than viruses isolated from control mice, suggesting that favipiravir can drive persistent MNV infections to extinction through the accumulation of debilitating mutations. We also showed that the antiviral activity of favipiravir is sustained during prolonged treatment periods (>50 days) without any observed rebound in viral titers. Our data also suggested that favipiravir is more efficient than ribavirin in the control of MNV infections in vivo, possibly because favipiravir induces more-effective increases in mutation frequency. Ribavirin and favipiravir are purine analogues that can be triphosphorylated within the cell and then incorporated into nascent viral genomes by the viral RdRp. Their mutagenic activity resides in their ambiguous pairing behavior, establishing stable base pairing with both uridine and cytidine. This ambiguity can occur as they are incorporated into the nascent viral RNA and in the next round of replication acting in the template strand, resulting altogether in an increase in the number of transition mutations.

The main advantage of lethal mutagenesis relative to other antiviral strategies is that it causes a continuous attenuation of the replicating virus, leading to clearance of infection. In the absence of the drug, attenuating deleterious mutations may remain imprinted in the viral population and in any transmitted virus. In addition, it has been demonstrated that significantly attenuated and defective variants generated during mutagenesis can interfere with infection by replication-competent viruses, thereby facilitating viral extinction [18]. These results raise the possibility of using favipiravir and lethal mutagenesis as an antiviral strategy to treat chronic HuNoV infections in immunocompromised individuals. A recent study has indicated that ribavirin may be effective in some chronically infected patients, although whether the antiviral activity observed was due to mutagenesis was not examined [19]. Clearly, further studies in this area are warranted.

Recent studies encourage the development of new combinational approaches involving a mutagenic compound and a classical inhibitor, to improve the efficacy of therapies based on lethal mutagenesis [18]. A possible limitation of such cocktails may be the greater likelihood of resistance to classical inhibitors as a consequence of the increased mutation frequencies. Alternative combinational approaches may involve sequential administration of inhibitor and mutagen to reduce the possibility of resistance emerging, a regimen recently shown to be more efficient than simultaneous administration [18]. Combinations of mutagens and inhibitors targeting critical host factors may also present a rational approach, with a lower risk of generating viral resistance.

## SUMMARY

In recent years, there has been significant progress in the development of small-molecule inhibitors for HuNoV (Table 1), driven by different approaches and accompanied by increased understanding of the norovirus life cycle and development of new systems for studying norovirus replication. Importantly, however, further studies will be required to bring any of these potential compounds through clinical trials and into the clinic. To date, the only reported preliminary clinical trial tested nitazoxanide, a compound licensed against certain parasites, which produced a modest decrease in symptom duration in healthy individuals but, more importantly, resolved symptoms within a single chronically infected patient [10]. However, no further use has been reported since 2011. Evaluating inhibitors that are currently in trials or under development for other viruses with conserved protein functions, including 2CMC derivatives and favipiravir, may serve to accelerate treatments for HuNoV through clinical trials.

**Table 1.** Summary of Potential Norovirus Antivirals

Compound	Target	Mechanism of Action
Classical inhibitor		
IFN-λ	Host cells	Induction of antiviral state, specific mediators unknown [11]
Rupintrivir	Viral protease	Irreversible inhibitor of active site [12]
2CMC	Viral polymerase	Nucleoside analogue [16]
Suramin	Viral polymerase	Nonnucleoside analogue [10]
NF203	Viral polymerase	Nonnucleoside analogue [10]
PPNDS	Viral polymerase	Nonnucleoside analogue [10]
Host cell inhibitor		
WP1130	Cellular deubiquitinases	Indirect activation of the unfolded protein response [16]
Chemical mutagen		
Favipiravir	Viral polymerase	Lethal mutagenesis [17]
Ribavirin	Viral polymerase	Lethal mutagenesis [17]

Abbreviations: 2CMC, 2′-C-methylcytidine; IFN-λ, interferon λ.

## Supplementary Data

Supplementary materials are available at http://jid.oxfordjournals.org. Consisting of data provided by the author to benefit the reader, the posted materials are not copyedited and are the sole responsibility of the author, so questions or comments should be addressed to the author.

Supplementary Data
