# Supplementary material for: Advances Toward a Norovirus Antiviral: From Classical Inhibitors to Lethal Mutagenesis
Source: J Infect Dis. 2015 Dec 19;213(Suppl 1):S27–31. doi: 10.1093/infdis/jiv280 (PMC4704654; doi:10.1093/infdis/jiv280)
Supplement: Supplementary Data [file supp_213_suppl-1_S27__index.html]

Supplementary Data 

# Advances Toward a Norovirus Antiviral: From Classical Inhibitors to Lethal Mutagenesis

## Supplementary Data

Supplementary Data

- Supplementary Data - Docx file
