# Supplementary material for: Advances Toward a Norovirus Antiviral: From Classical Inhibitors to Lethal Mutagenesis
Source: J Infect Dis. 2015 Dec 19;213(Suppl 1):S27–31. doi: 10.1093/infdis/jiv280 (PMC4704654; doi:10.1093/infdis/jiv280)
Supplement: Supplementary Data [file supp_jiv280_jiv280supp.docx]

**Supplementary references**

1. Payne DC, Vinje J, Szilagyi PG, et al. Norovirus and medically attended gastroenteritis in U.S. children. N Engl J Med 2013; 368:1121-30.

2. Harris JP, Adams NL, Lopman BA, Allen DJ, Adak GK. The development of Web-based surveillance provides new insights into the burden of norovirus outbreaks in hospitals in England. Epidemiol Infect 2014; 142:1590-8.

3. Ronchetti AM, Henry B, Ambert-Balay K, et al. Norovirus-related chronic diarrhea in a patient treated with alemtuzumab for chronic lymphocytic leukemia. BMC Infect Dis 2014; 14:239.:10.1186/471-2334-14-239.

4. Woodward JM, Gkrania-Klotsas E, Cordero-Ng AY, et al. The role of chronic norovirus infection in the enteropathy associated with common variable immunodeficiency. Am J Gastroenterol 2015; 110:320-7.

5. Patel MM, Widdowson,M-A, Glass,R.I., Akazawa,K., Vinjé,J., Parashar,U.D. . Systematic literature review of role of noroviruses in sporadic gastroenteritis. Emerg Infect Dis 2008; 14:1224-31.
